# Assessing the effects of tempol on renal fibrosis, inflammation, and oxidative stress in a high-salt diet combined with 5/6 nephrectomy rat model: utilizing oxidized albumin as a biomarker

**DOI:** 10.1186/s12882-024-03495-0

**Published:** 2024-02-23

**Authors:** Beibei Liu, Yanling Hu, Danyang Tian, Jianlong Dong, Bing-Feng Li

**Affiliations:** 1 College of Life and Health, Nanjing Polytechnic Institute, No.188 Xinle Road, Luhe District, 210048 Nanjing, Nanjing, Jiangsu China; 2 Department of Physiology, Hebei Medical University, Shijiazhuang, China; 3 Hebei University of Chinese Medicine, The First Affiliated Hospital, Shijiazhuang, China

**Keywords:** Tempol, Oxidative stress, 5/6 nephrectomy, Oxidized albumin, Chronic kidney disease

## Abstract

**Background:**

Oxidative stress has been implicated in the pathogenesis of chronic kidney disease (CKD), prompting the exploration of antioxidants as a potential therapeutic avenue for mitigating disease progression. This study aims to investigate the beneficial impact of Tempol on the progression of CKD in a rat model utilizing oxidized albumin as a biomarker.

**Methods:**

After four weeks of treatment, metabolic parameters, including body weight, left ventricle residual weight, kidney weight, urine volume, and water and food intake, were measured. Systolic blood pressure, urinary protein, oxidized albumin level, serum creatinine (Scr), blood urea nitrogen (BUN), 8-OHdG, TGF-β1, and micro-albumin were also assessed. Renal fibrosis was evaluated through histological and biochemical assays. P65-NF-κB was quantified using an immunofluorescence test, while Smad3, P65-NF-κB, and Collagen I were measured using western blot. TNF-α, IL-6, MCP-1, TGF-β1, Smad3, and P65-NF-κB were analyzed by RT-qPCR.

**Results:**

Rats in the high-salt diet group exhibited impaired renal function, characterized by elevated levels of blood urea nitrogen, serum creatinine, 8-OHdG, urine albumin, and tubulointerstitial damage, along with reduced body weight. However, these effects were significantly ameliorated by Tempol administration. In the high-salt diet group, blood pressure, urinary protein, and oxidized albumin levels were notably higher compared to the normal diet group, but Tempol administration in the treatment group reversed these effects. Rats in the high-salt diet group also displayed increased levels of proinflammatory factors (TNF-α, IL-6, MCP1) and profibrotic factors (NF-κB activation, Collagen I), elevated expression of NADPH oxidation-related subunits (P65), and activation of the TGF-β1/Smad3 signaling pathway. Tempol treatment inhibited NF-κB-mediated inflammation and TGF-β1/Smad3-induced renal fibrosis signaling pathway activation.

**Conclusion:**

These findings suggest that Tempol may hold therapeutic potential for preventing and treating rats undergoing 5/6 nephrectomy. Further research is warranted to elucidate the mechanisms underlying Tempol’s protective effects and its potential clinical applications. Besides, there is a discernible positive relationship between oxidized albumin and other biomarkers, such as 8-OHG, urinary protein levels, mALB, Scr, BUN, and TGF-β1 in a High-salt diet combined with 5/6 nephrectomy rat model. These findings suggest the potential utility of oxidized albumin as a sensitive indicator for oxidative stress assessment.

**Supplementary Information:**

The online version contains supplementary material available at 10.1186/s12882-024-03495-0.

## Introduction

Chronic kidney disease (CKD) stands as a significant contributor to human health, ultimately culminating in end-stage renal disease (ESRD), characterized by a progressive and irreversible loss of renal function [1]. CKD exerts a global impact, affecting millions of individuals worldwide and correlating with substantial morbidity and mortality [2]. Oxidative stress emerges as a pivotal feature of CKD, playing a crucial role in the development of its complications [3–5]. While reactive oxygen species (ROS) play an indispensable role in normal cellular functions, an excessive accumulation of ROS in tissues under pathological conditions results in oxidative stress, triggering deleterious outcomes such as inflammation and fibrosis [6–9]. Previous research has substantiated an elevated level of oxidized albumin in both patients and animal models with CKD [10–13]. ROS activation of the redox-sensitive epidermal growth factor receptor (EGFR) emerges as a critical mechanism, serving as the primary transcription factor for adhesion molecules and proinflammatory cytokines, thereby promoting inflammation in CKD [14, 15]. The concentrations of inflammatory biomarkers, including TNF-α and IL-6, along with leukocyte counts, exhibit an increase in CKD patients; however, their specific roles as drivers or consequences of disease progression remain unclear.

Current treatments for chronic kidney disease (CKD) are notably limited, emphasizing the need for the development of novel therapies that target crucial pathways. Tempol, a chemical compound synthesized through the oxidation of the stable free radical 4-hydroxy-2,2,6,6-tetramethyl-piperidin-1-oxyl, has emerged as a promising candidate [4, 16]. Research indicates that Tempol effectively ameliorates endothelial dysfunction in diabetic rats and demonstrates a capacity to reduce both cell proliferation and matrix accumulation in the kidneys [17, 18]. Moreover, Tempol has been validated for its ability to lower proteinuria levels in diabetic nephropathy patients [19, 20]. Beyond these benefits, Tempol exhibits inhibitory effects against coagulation, inflammation, and oxidation, in addition to its capacity to regulate blood lipids. However, the comprehensive impact of Tempol on renal fibrosis, inflammation, and oxidative stress remains incompletely understood. Given the crucial role of these functions in the progression of chronic renal failure (CRF), our study aimed to thoroughly investigate the effectiveness of Tempol as an intervention in a rat 5/6 nephrectomy model.

The 5/6 nephrectomy (5/6 Nx) rat model is extensively employed for the investigation of progressive chronic kidney disease (CKD) [13, 21, 22]. This model effectively replicates clinical CKD, where diminished renal mass prompts a compensatory augmentation in the remaining kidney, subsequently leading to progressive renal fibrosis, inflammation, and oxidative stress [9, 18, 23, 24]. Studies utilizing this model can offer valuable insights into the underlying mechanisms and the potential for therapeutic interventions.

In this study, our primary objective was to examine the impact of Tempol on renal fibrosis, oxidative stress, and inflammation in 5/6 nephrectomy rats utilizing oxidized albumin as a biomarker. Our hypothesis posited that Tempol treatment would mitigate renal fibrosis, oxidative stress, and inflammation, thereby decelerating the progression of CKD. By investigating the potential therapeutic advantages of Tempol in the 5/6 nephrectomy model, our findings may contribute to the formulation of innovative treatments for this debilitating condition. Besides, the positive relationship between oxidized albumin and other biomarkers suggests the potential utility of oxidized albumin as a sensitive indicator for oxidative stress assessment.

## Materials and methods

### Animals

Three-week-old male Sprague Dawley (SD) rats (40 gram to 50 gram) obtained from the Experimental Animal Center of Yangzhou University underwent 5/6 nephrectomy. Then they were housed under a relatively humidity (60%±5%) at comparatively low temperature (23 ± 1.5 °C). The animals were maintained on a twelve-hour light-dark cycle, allowing them unrestricted access to both water and food. After four weeks of treatment, blood samples were collected via cardiac puncture. This study was conducted in compliance with the ethical guidelines set by Laboratory Animal Ethical and Welfare Committee of Hebei Medical University, with the ethical approval number IACUC-Hebmu-2,022,042.

### Experimental protocol

Rats (*n* = 24) were randomly divided into four groups: (1) the blank group (no 5/6 nephrectomy + 0.3% NaCl diet, *n* = 6), (2) the normal diet group (5/6 nephrectomy + 0.3% NaCl, *n* = 6), (3) the high-salt group (5/6 nephrectomy + 8% NaCl, *n* = 6), and (4) the treatment group (5/6 nephrectomy + 8% NaCl + Tempol in drinking water with a concentration of 1 mmol/kg/day, *n* = 6)(Tempol, 4-hydroxy-2,2,6,6-tetramethyl-piperidine-N-oxyl). Rats that did not survive post-operation (*n* = 3) were excluded from subsequent analysis. Tempol treatment was administered from week 3 until sacrifice (week 7). The rats were sacrificed at week 7. Rats were anesthetized with pentobarbital sodium (36–39 mg/kg body weight) by intraperitoneal injection to relieve painfulness. Systolic blood pressure (SBP) and body weight (BW) were measured prior to sacrifice, utilizing a noninvasive computerized tail-cuff manometry system. The assessment of urinary protein involved collecting 24-hour urine specimens in metabolic cages after 4 weeks of treatment. Urinary protein was measured by collecting 24-hour urine specimens in metabolic cages after 4 weeks of treatment. Kidney tissues and blood samples were collected for subsequent analysis. All analyses were performed blindly.

### Metabolic testing

Prior to sacrifice, the following parameters were measured: body weight (g), 24-hour drinking water (ml/24 h), urine volume (ml/24 h), eating amount (mg/24 h), residual kidney amount (mg), residual left ventricular amount (mg) and systolic blood pressure.

### Oxidized albumin measurement

Serum samples were thawed from a -80 °C freezer for analysis. The oxidized albumin level was measured using High-Performance Liquid Chromatography (HPLC) with fluorescence detection at wavelengths of 280 and 340 nm. Two eluent buffers, consisting of 60 mM sodium sulfate and 25 mM phosphoric acid buffer, were employed. Three microliters of serum samples were analyzed at a flow rate of 1 ml/min and an oven temperature of 40 °C. The results were calculated using the equation: Oxidized albumin% = (Oxidized albumin / (Reduced albumin + Oxidized albumin)) × 100%.

### Biochemical testing

Blood samples were collected and centrifuged at 3000 rpm (4 °C, 5 min) to separate serum, which was then stored at -80 °C for analysis. In addition to testing serum oxidized albumin, the collected serum samples were also utilized for the analysis of creatinine, BUN, 8-OHdG, and TGF-β1. Serum creatinine (Scr) level was determined using the sarcosine oxidase technique, while the urease and glutamate dehydrogenase enzymatic reaction method was used for BUN level testing. For urine analysis, the supernatant of urine samples was centrifuged at 3500 g (24 °C) for 15 min. Rat urine collected from a metabolic cage for 24 h was measured for volume using a volumetric canister, and urine protein concentration was determined using a specific kit (cat. no. MM-70785R1, MEIMIAN Bioengineering Institute) designed for urine protein analysis.

### Histology, immunohistochemistry, and immunofluorescence staining

The kidney tissues were harvested, incubated, and fixed in 4% paraformaldehyde for over 24 h at room temperature. Afterward, the samples were embedded in paraffin wax. Kidney Sect. (4 μm thick) underwent hematoxylin and eosin (H&E) staining, Masson’s staining, and immunofluorescent staining. For H&E and Masson’s staining, the nuclei were stained with hematoxylin for 3 min at 37˚C, Ponceau for 3 min, toluidine blue for 3 min, and 0.5% eosin for 30 s. Immunohistochemical staining was performed using antibodies targeting α smooth muscle actin (α-SMA) (cat. no. 19,245 S; Cell signal). The sections were blocked with 3% hydrogen peroxide for 20 min at 37˚C, and primary antibodies (2% normal goat serum, cat. no. BL003A; Biosharp; 1:6000) were incubated overnight at 4˚C. Subsequently, sections were incubated at 37˚C with a secondary antibody derived from normal goat serum (1:6000; cat. no. BL003A; Biosharp) for 1 h, followed by color development using DAB (cat. no. ZLI 9018; ZSGB BIO) staining solution at room temperature. The sections were observed under a light photomicrograph device (Leica; DMi8) at x400 or x200 magnification. For immunofluorescence staining, sections were blocked with PBS containing 5% BSA (cat. no. 4240GR500; BioFROXX) for 15 min at 37˚C, and primary antibodies were incubated overnight at 4˚C in the dark. Finally, sections were incubated at 37˚C with PBS containing 2% BSA and diluted secondary antibody (1:2000; cat. no. 4240GR500; BioFROXX) for 1 h. Hoechst (cat. no. H33342; Biosharp) staining solution was used for color development for 10 min at room temperature. The sections were visualized using scanning microscopy (Leica; DMi8) at x400 magnification. The staining area was quantified in 10 consecutive high-power fields and expressed as the number of positive views or percentage of positive area.

### Western blot analysis

In vivo experiments: Cardiac tissue was homogenized in RIPA buffer for protein extraction. Western blotting was performed using SDS-PAGE and PVDF membranes. Total protein was extracted from kidney tissue using lysis buffer (cat. no. BL504A; Biosharp). The protein concentration was determined using a bicinchoninic acid kit (cat. no. BL521A; Biosharp). Histone concentration was adjusted to 5 mg/ml. Each sample was loaded onto a 12% gel for western blotting, with 30 µg per lane, and transferred to PVDF membranes. Following blocking with 5% BSA at room temperature for 1 h, PVDF membranes were incubated overnight at 4˚C with primary antibodies against Smad3(1:1000, cat. no. ab227223; Abcam), P65-NF-ΚB (1:500, cat. no. 10745-1-AP; Proteintech), and TGF-β1(1:1000, cat. no. 21**8-1-AP; Proteintech). PVDF membranes were incubated with corresponding secondary antibodies at room temperature for one hour. Protein bands were visualized using the ECL Chemiluminescence Apparatus (BIO-RAD, ChemiDocXRS+), and protein expression was quantified using ImageJ with GAPDH as the loading control.

### Reverse transcription quantitative (RT-q) PCR

Total RNA was extracted from tissue samples using HI ScriptIIQ RT Super Mix for qPCR (Vazyme; R222). The RNA was converted into cDNA using 2×Q3 SYBR qPCR Master mix (Universal) (CRONDABIO; KCD-M1004) through reverse transcription. RT-qPCR was performed on the Cobas z 480 Real Time PCR System (Light Cycler 480II; Roche) using SYBR Green I as the detection fluorophore according to the manufacturer’s instructions. The thermocycling protocol consisted of initial denaturation at 95˚C for 2 minutes, followed by 45 cycles of 10 seconds at 95˚C and 20 seconds at 60˚C. The mRNA levels of IL6, MCP1, TNFα, TGFβ1, Smad3, and β-actin were quantified using the following primer sets: IL6 forward 5’ TTGCCTTCTTGGGACTGA 3’ and reverse 5’ TTGCCATTGCACAACTCTT 3’; MCP1 forward 5’ CACGCTTCTGGGCCTGTT 3’ and reverse 5’ CCGACTCATTGGGATCATCTT 3’; TNFα forward 5’ GGCCACCACGCTCTTCTGTC 3’ and reverse 5’ TGGGCTACGGGCTTGTCACTC 3’; TGFβ1 forward 5’ GCAACAATTCCTGGCGTTACCTT 3’ and reverse 5’ CACCTCGACGTTTGGGACTGATC 3’; Smad3 forward 5’ GAGGAGAAGTGGTGCGAGAAGG 3’ and reverse 5’ CCGTAACTCATGGTGGCTGTGC 3’; β-actin forward 5’ TGTGACGTTGACATCCGTAAAGACC 3’ and reverse 5’ TGCTAGGAGCCAGGGCAGTAA 3’. The primers for IL6, MCP1, TNFα, TGFβ1, Smad3, and β-actin were supplied by Sangon Biotech Co., Ltd. The primers for IL-1β and TNF-α were supplied by Abcam. The mRNA levels were measured using the 2-△△CT approach and standardized to the internal reference gene β-actin.

### Statistical analysis

The data is expressed as mean ± SEM. One-way ANOVA followed by Tukey’s post hoc test was applied for multiple group comparisons. Statistical significance was considered at *p* < 0.05, indicating significant results for all findings.

## Results

### Effects of high-salt diet on physiological parameters and the protective role of tempol in rats

**Fig. 1:**
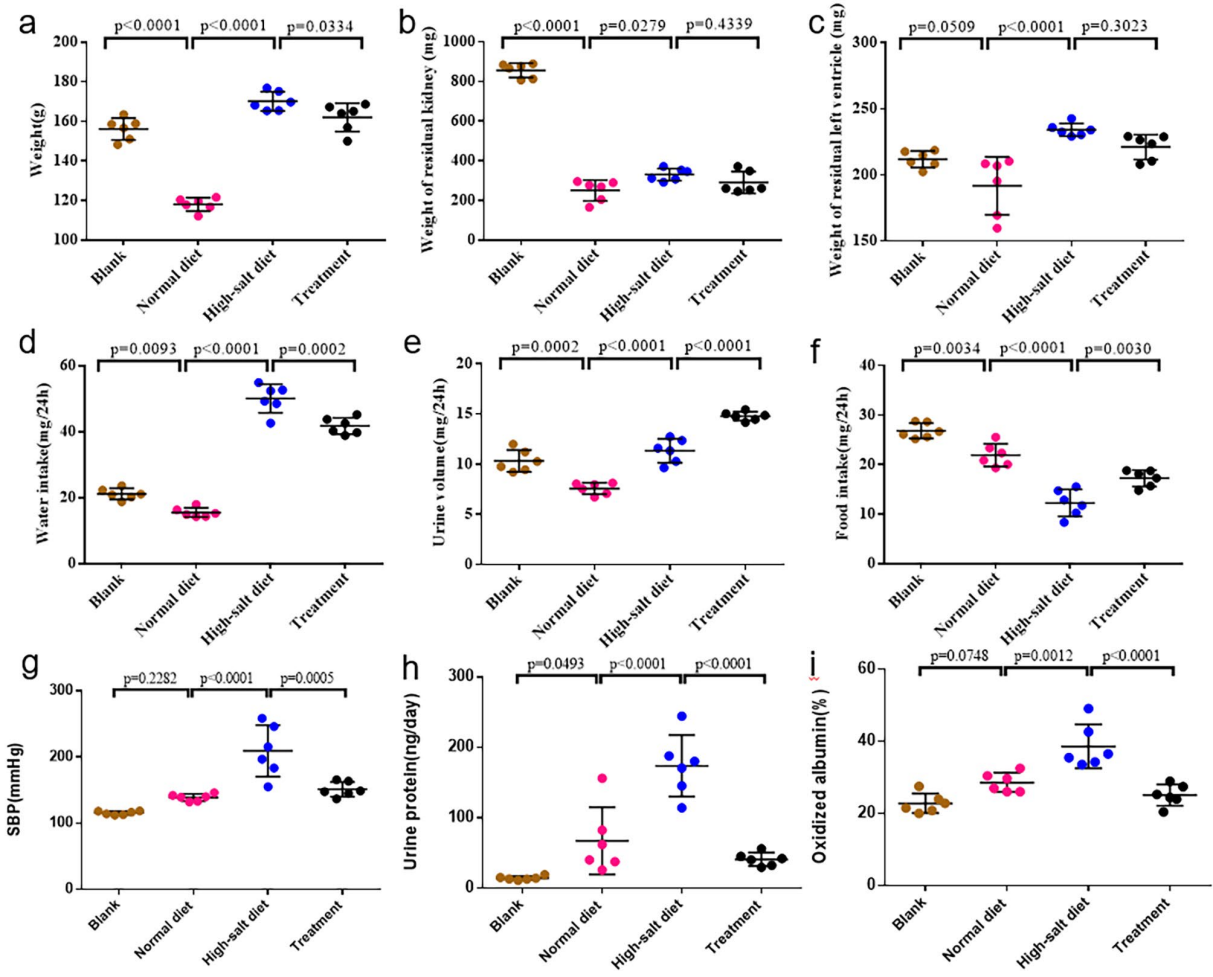
Physiological parameters and oxidized albumin validation study of rats. **(a)**Body weight. Rats in high-salt diet group had increased body weight compared to normal-salt group. **(b)**Weight of residual kidney. Weight of residual kidney showed no significant difference among groups after 4-week treatment. **(c)**Weight of residual left ventricle. weight of residual left ventricle was higher in high-salt group rats compared to normal-salt group rats. **(d)**Water intake. High-salt group rats exhibited significantly higher water intake compared to normal-salt group rats. **(e)**Urine volume. High-salt group rats exhibited significantly higher urine volume compared to normal-salt group rats. **(f)**Food intake. High-salt group rats exhibited significantly lower food intake compared to normal-salt group rats. **(g)**Systolic Blood Pressure. Rats in high salt diet group had significantly elevated systolic blood pressure compared to normal salt group. Tempol loading improved systolic blood pressure. **(h)**Urinary Protein. After high-salt treatment, urinary protein level in high-salt diet group was significantly higher than that in normal-salt diet group. After Tempol loading, urinary protein level decreased. **(i)**Oxidized Albumin. The high-salt diet group had significantly higher serum oxidized albumin level compared to normal-salt diet group. Administration of Tempol effectively reversed the impact of high salt loading, resulting in reduced serum oxidized albumin level.The values are shown as AVE ± SEM

Rats in the high-salt diet group exhibited an increased body weight compared to the normal-salt group (170.2 ± 4.9 g vs. 118.1 ± 5.1 g, Fig. 1a). The weight of the residual kidney showed no significant differences among the groups after the 4-week treatment (Fig. 1b). However, the weight of the residual left ventricle was higher in rats from the high-salt group compared to those from the normal-salt group (330.0 ± 35.5 mg vs. 250.0 ± 73.5 mg, Fig. 1c). Rats in the high-salt group demonstrated significantly higher water intake (Fig. 1d) and urine volume (Fig. 1e), along with lower food intake (Fig. 1f), compared to their counterparts in the normal-salt group.

As anticipated, rats subjected to a high-salt diet group exhibited a significantly elevated systolic blood pressure compared to their counterparts in the normal-salt group (209.4 ± 39.0 mmHg vs. 138.8 ± 5.4 mmHg, Fig. 1g). Introduction of Tempol resulted in a notable amelioration of systolic blood pressure (151.3 ± 11.2 mmHg vs. 209.4 ± 39.0 mmHg, Fig. 1g). Following high-salt treatment, the urinary protein level in the high-salt diet group was significantly higher than that in the normal-salt diet group (173.7 ± 43.8 ng/day vs. 67.3 ± 47.9 ng/day). Subsequent administration of Tempol resulted in a decrease in urinary protein levels (40.9 ± 9.4 ng/day vs. 173.7 ± 43.8 ng/day, Fig. 1h). The high-salt diet group exhibited a significantly higher serum oxidized albumin level compared to the normal-salt diet group (38.6%±6.1% vs. 28.6%±2.7%, Fig. 1i). Administration of Tempol effectively reversed the impact of high salt loading, leading to a reduced serum oxidized albumin level (25.1%±2.9% vs. 38.6%±6.1%, Fig. 1i).

### Effects of high-salt diet on biochemical testing parameters and the antioxidant effect of tempol

**Fig. 2:**
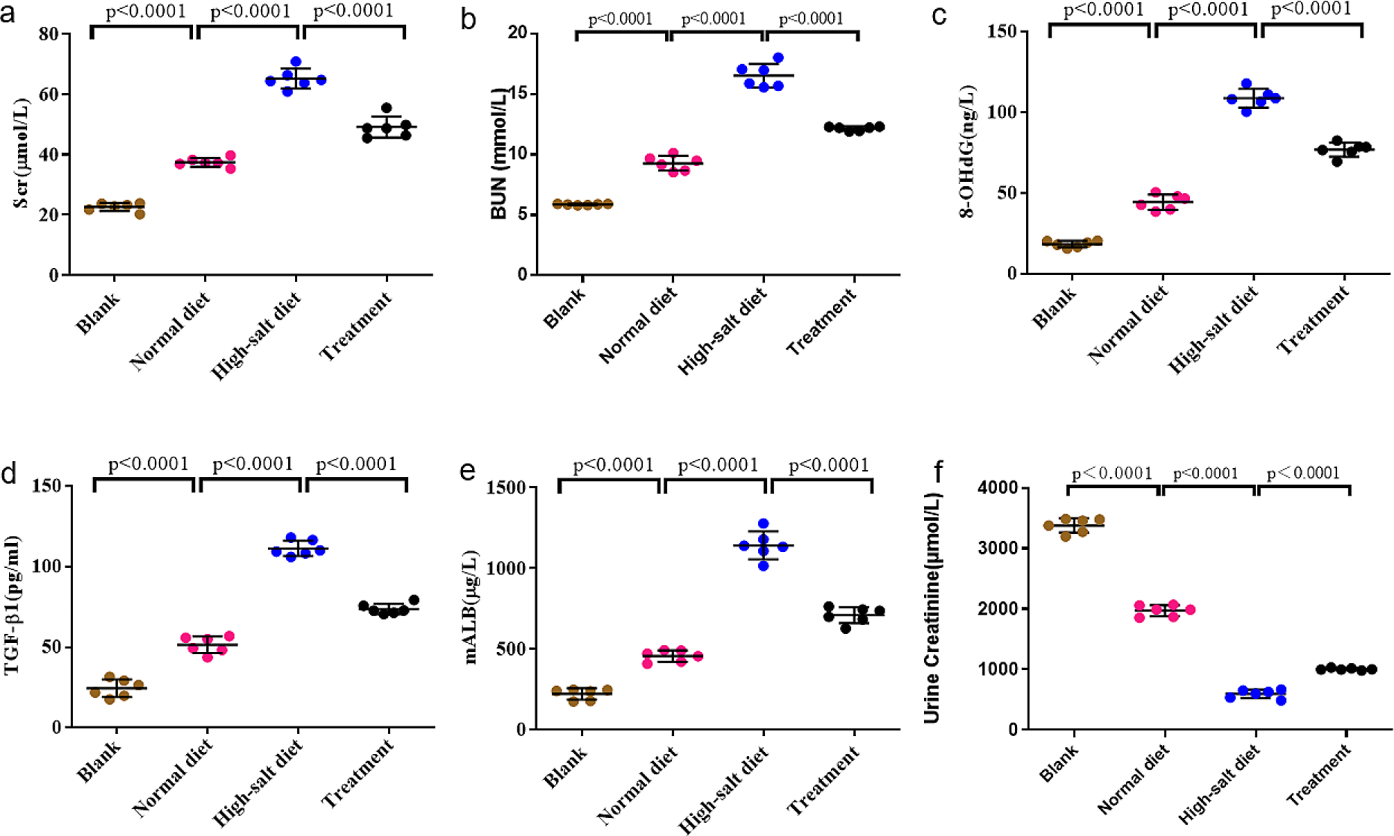
Biochemical testing parameters. **(a)(b)**Serum creatinine and BUN. Serum creatinine and BUN levels were higher in rats fed a high-salt diet compared to those on a normal-salt diet. However, rats in the Treatment group had lower serum creatinine and BUN levels compared to the high-salt diet group. **(c)** 8-OHdG. The high-salt diet also led to increased serum 8-OHdG levels, but Tempol reversed this effect. **(d) (e) (f)** TGF-β1, mALB and urine creatinine. Administering Tempol improved oxidative stress in rats on a high-salt diet, as evidenced by normalized levels of TGF-β1, and mALB. However, these levels were still higher than in rats on a normal-salt diet. The values are shown as AVE ± SEM

Serum creatinine (Fig. 2a) and blood urea nitrogen (BUN) levels (Fig. 2b) exhibited elevation in rats subjected to a high-salt diet compared to those on a normal-salt diet. However, the Treatment group displayed diminished levels of lower serum creatinine and BUN levels compared to the High-salt diet group. The high-salt diet also led to increased serum 8-OHdG levels (Fig. 2c), but Tempol reversed this effect. The introduction of Tempol ameliorated oxidative stress in rats on a high-salt diet, as evidenced by normalized levels of oxidized albumin, 8-OHdG, TGF-β1(Fig. 2d), mALB (Fig. 2e) and urine creatinine (Fig. 2f). However, these levels remained higher than those in rats on a normal-salt diet.

### Morphological and immunostaining analysis of renal damage induced by high-salt diet and the protective effect of tempol

**Fig. 3:**
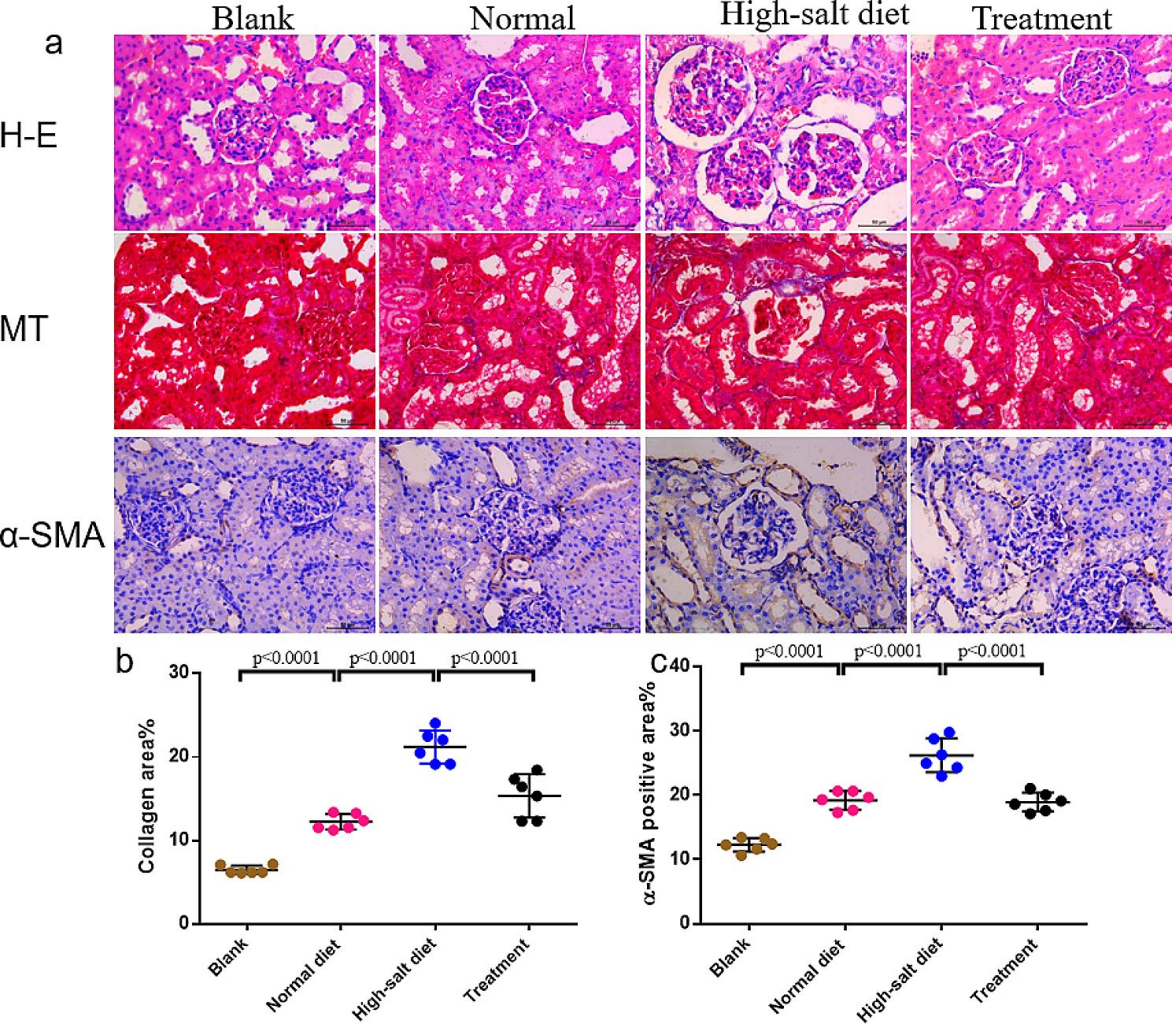
Histology Findings. **(a)**In the High-salt diet group, significant morphological changes were observed in histological examination, including glomerular hypertrophy and diffuse atrophy of renal tubular epithelial cells. **(b)**The Treatment group showed improvement compared to the High-salt diet group, but still had higher damage than the Normal salt group, characterized by glomerular lobulation and an increased glomerular cystic cavity. **(c)** In the Blank group, expression is mainly in the renal tubular wall, with minimal occurrence in the interstitium. In both the Normal diet and Treatment groups, positive expression predominantly occurs in the renal tubular wall, with minimal presence in the interstitium. The High-salt diet group exhibits noticeable expression in the renal interstitium. The values are shown as AVE ± SEM

In the High-salt diet group, significant morphological changes were observed in histological examination, including glomerular hypertrophy and diffuse atrophy of renal tubular epithelial cells (Fig. 3a). These changes indicate that the interstitium, tubules, and glomeruli in the Normal salt group remained morphologically unaffected. The Treatment group exhibited improvement compared to the High-salt diet group, but still displayed higher damage than the Normal salt group, characterized by glomerular lobulation and an increased glomerular cystic cavity.

In terms of collagen staining, the Blank group showed minimal blue staining of collagen fibers. (Fig. 3b) The Normal salt group and Treatment groups showed some blue staining in the glomerular cystic cavity, but not in other areas. In contrast, the High-salt diet group exhibited a larger distribution of blue-stained collagen in the interstitium.

Concerning the area positive for α-SMA, the predominant expression in the Blank group is primarily localized to the renal tubular wall, with negligible expression in the renal interstitium. In both the Normal diet and Treatment groups, positive expression is predominantly observed in the renal tubular wall, with minimal occurrence in the interstitium. In the High-salt diet group, expression is noticeably present in the renal interstitium (Fig. 3c).

**Fig. 4:**
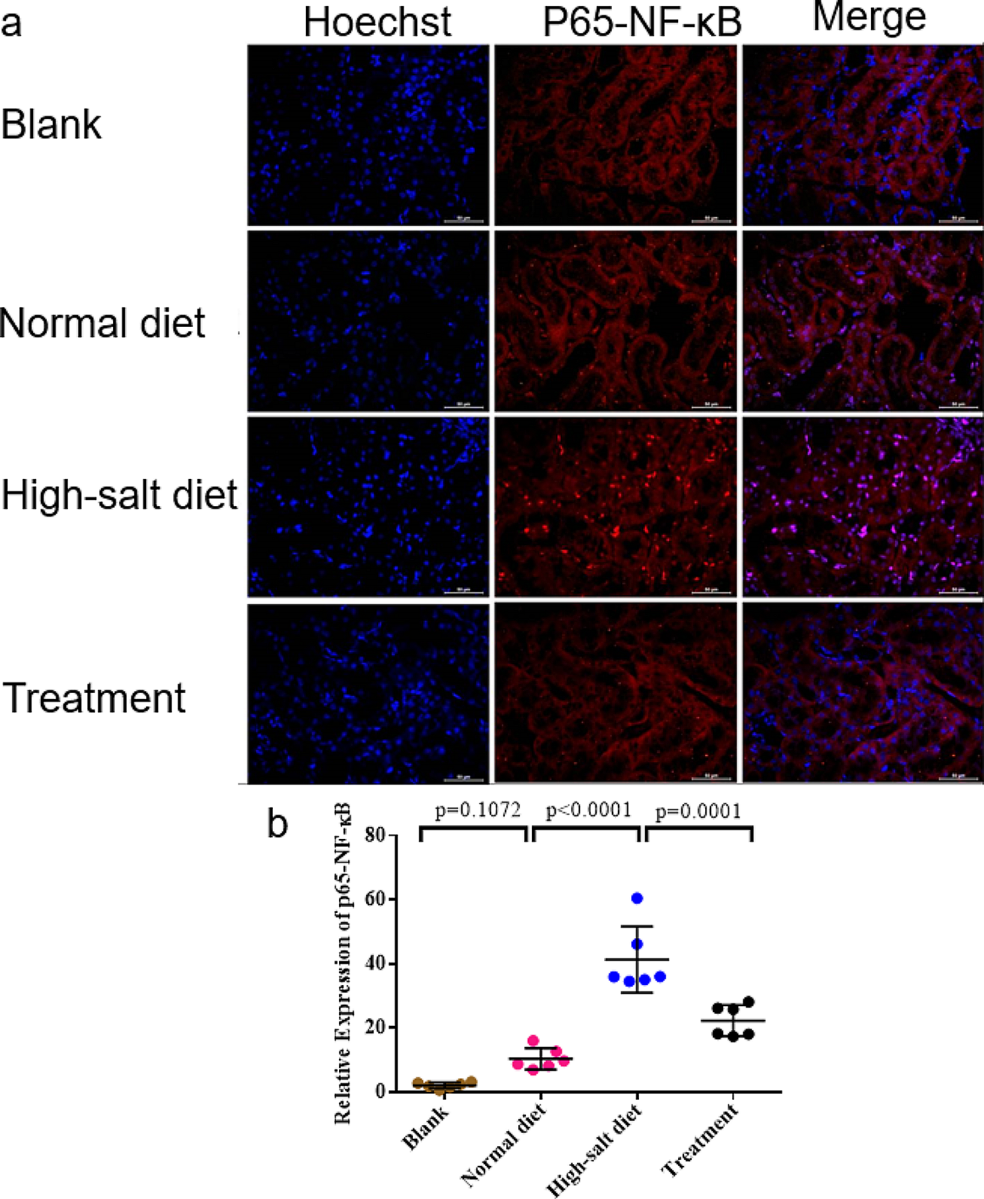
Immunohistochemistry, and immunofluorescence staining findings. **(a)** Immunostaining analysis showed the presence of p65-NF-κB (red fluorescence) in the High-salt diet group. **(b)**Treatment with Tempol resulted in a notable decrease in the nuclear localization of p65-NF-κB. Additionally, the normal diet group had lower levels of nuclear p65-NF-κB compared to the treatment group. The values are shown as AVE ± SEM

Immunostaining analysis revealed the presence of p65-NF-κB (red fluorescence) in the High-salt diet group. Treatment with Tempol resulted in a notable decrease in the nuclear localization of p65-NF-κB. Additionally, the normal diet group exhibited lower levels of nuclear p65-NF-κB compared to the treatment group. Refer to Fig. 4 for visual representation.

### Protein expression levels of Smad3, TGF-β1, p65-NF-κB, and collagen I: implications for renal damage and the protective effect of tempol

**Fig. 5:**
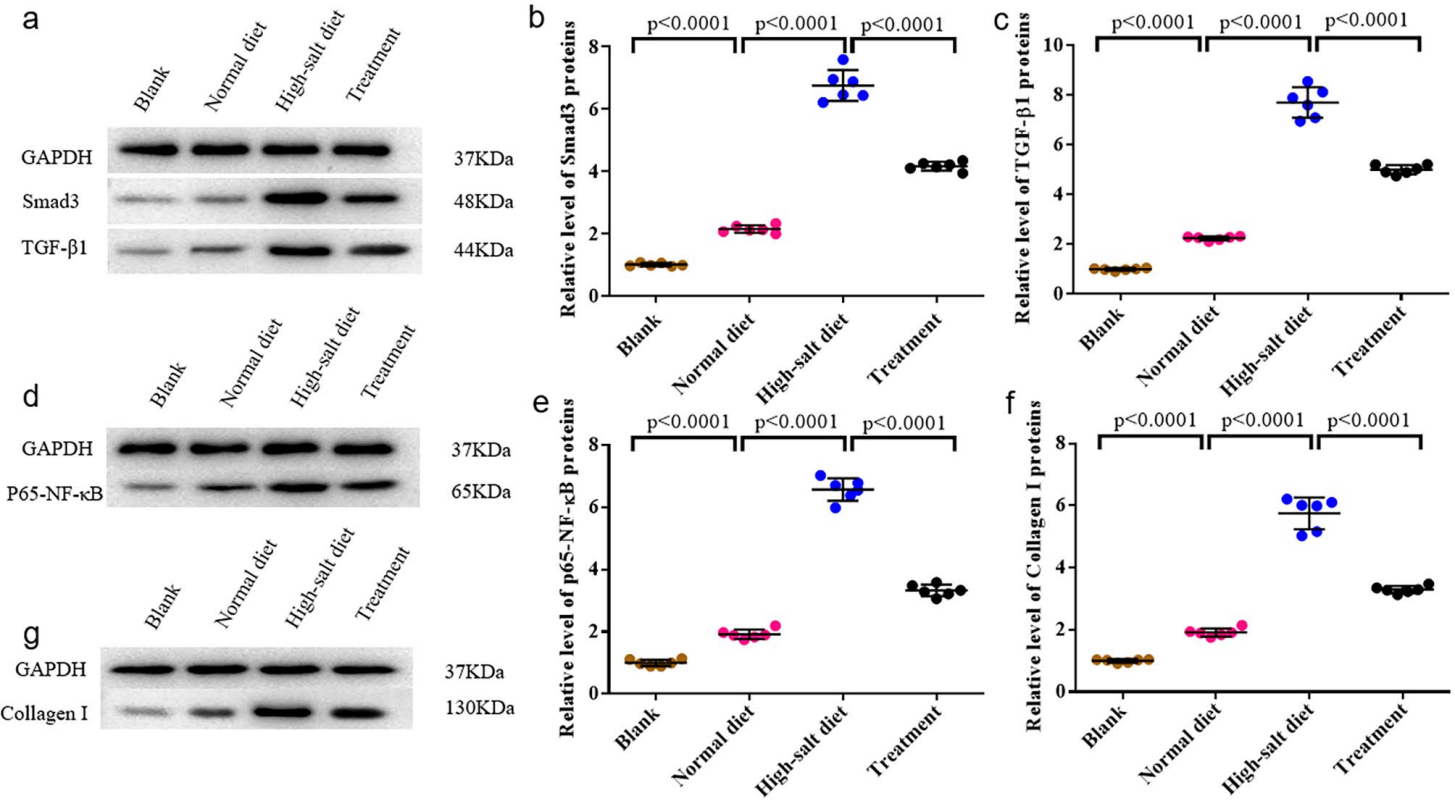
Western blot testing. **(a)**Representative western blots of Smad3/GAPDH, TGF-β1/GAPDH. **(b)**Quantitative analysis of Smad3. **(c)**Quantitative analysis of TGF-β1. Compared to the Blank group, Smad3 protein and TGF-β1 protein expression in the other three groups were elevated, with the highest expression in the high-salt diet group followed by the treatment group, and the normal diet group had lower expression than the treatment group. **(d)**Representative western blots of P65-NF-κB/GAPDH. **(e)**Quantitative analysis of P65-NF-κB. Compared with the Blank group, the expression of p65-NF-κB protein was increased in the remaining three groups, with the highest expression in high salt diet group, followed by the treatment group, and the Normal diet group was lower than the treatment group. **(f)** Quantitative analysis of Collagen I. **(g)**Representative western blots of Collagen I/GAPDH. Compared to the Blank group, the expression of Collagen I protein was increased in the other three groups, with the highest expression in the high salt diet group, treatment with Tempol significantly ameliorated Collagen I protein expression in high-salt diet group rats although still higher than normal-salt diet group rats. The values are shown as AVE ± SEM

We measured the protein levels of Smad3(Fig. 5a), TGF-β1(Fig. 5a), p65-NF-κB(Fig. 5d) and Collagen I(Fig. 5g) in rats.

The expression trends of Smad3 protein and TGF-β1 protein were consistent among the groups. Compared to the Blank group, both Smad3 protein (Fig. 5b) and TGF-β1 protein expression (Fig. 5c) in the other three groups were increased, with the highest expression in the high-salt diet group followed by the treatment group, and the normal diet group displayed lower expression than the treatment group.

In contrast, the expression of p65-NF-κB protein increased in the remaining three groups compared to the Blank group. The high-salt diet group exhibited the highest expression, followed by the treatment group, with the normal diet group showing lower expression (Fig. 5e).

Furthermore, Collagen I protein expression increased in the other three groups compared to the Blank group, reaching the highest levels in the high-salt diet group. Treatment with Tempol significantly ameliorated Collagen I protein expression in high-salt diet group rats, although the levels remained still higher than normal-salt diet group rats (Fig. 5f).

### Effects of tempol treatment on inflammatory cytokine secretion and gene expression after high-salt diet

**Fig. 6:**
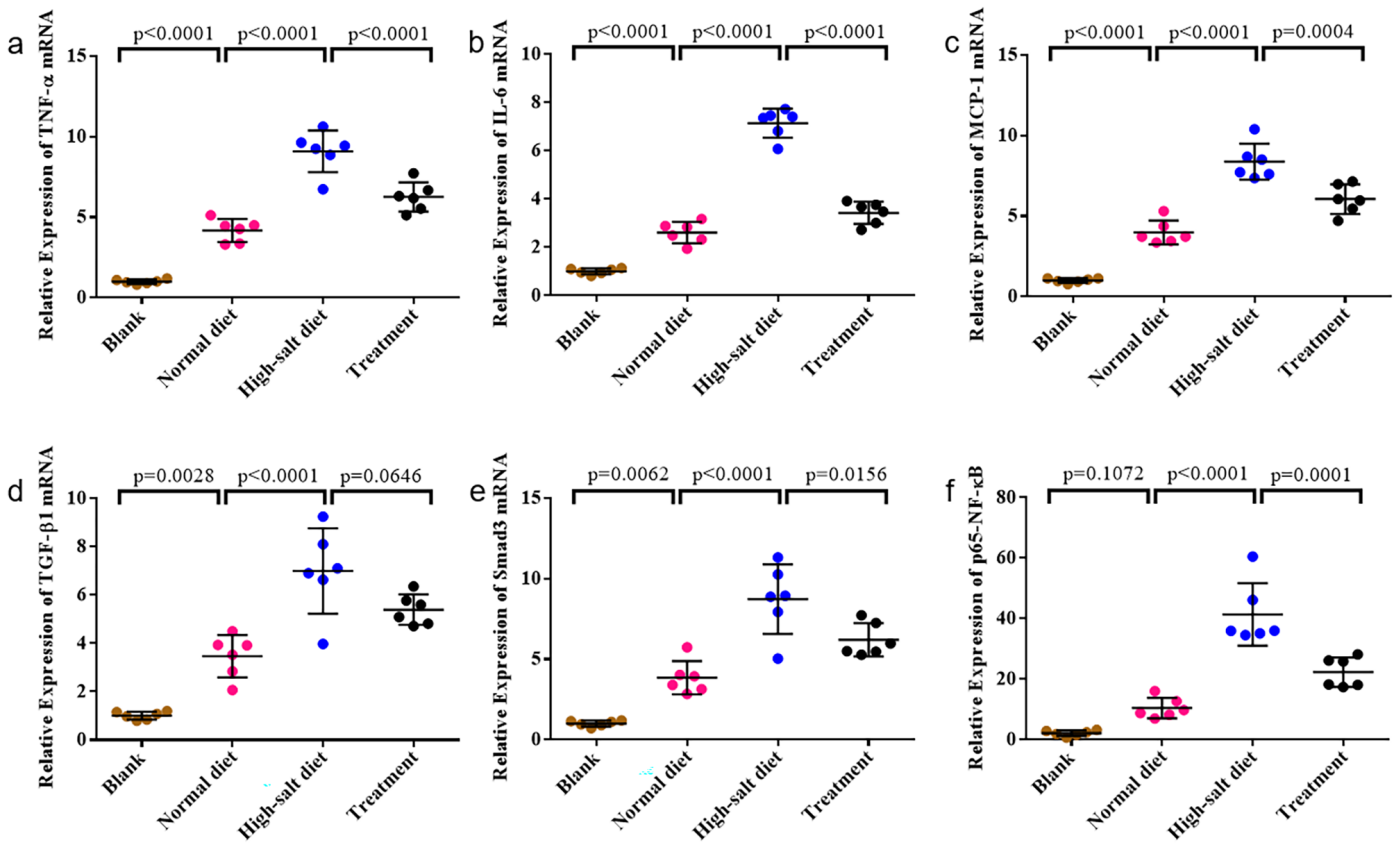
Tempol attenuates renal inflammation and renal fibrogenic pathways in high-salt diet group. Real-time PCR was employed to analyze the gene expression of TNF-α, IL-6, and MCP-1 in rats. TNF-α**(a)**, IL-6 **(b)** and MCP-1**(c)** expression were significantly attenuated by Tempol treatment following a notable increase in mRNA level induced by high salt loading. In the High-salt diet group of rats, renal TGF-β1**(d)**, Smad3**(e)** and p65-NF-κB**(f)** mRNA levels were significantly increased compared to Normal diet rats. However, in Treatment group, renal TGF-β1, Smad3 and p65-NF-κB mRNA levels were notably decreased compared to High-salt diet group. The values are shown as AVE ± SEM

Following the administration of a high-salt diet, a significant increase in the secretion of pro-inflammatory cytokines TNF-α, IL-6, and MCP-1 was observed. Real-time PCR was employed to analyze the gene expression of TNF-α, IL-6, and MCP-1 in rats. The expression of TNF-α(Fig. 6a), IL-6 (Fig. 6b) and MCP-1(Fig. 6c) was significantly attenuated by Tempol treatment following a notable increase in mRNA level induced by high salt loading.

In the High-salt diet group of rats, renal TGF-β1(Fig. 6d) and Smad3(Fig. 6e) mRNA levels were significantly increased compared to Normal diet rats. However, in Treatment group, renal TGF-β1 and Smad3 mRNA levels were notably decreased compared to High-salt diet group.

The p65-NF-κB mRNA level was notably elevated in high salt diet group compared to Normal salt group, and its expression was decreased by Tempol loading, as indicated in Fig. 6f. In accordance with the Western blot results, the immunostaining results demonstrated the presence of p65 (red fluorescence) in high salt diet group (Fig. 4a). Moreover, High-salt diet group exhibited an upregulation of p65-NF-κB expression in response to high salt diet stimulation, while a downregulation was observed in the Treatment group (*p* < 0.001) (Fig. 4b).

### Positive correlation between oxidized albumin and renal function indicators

**Fig. 7:**
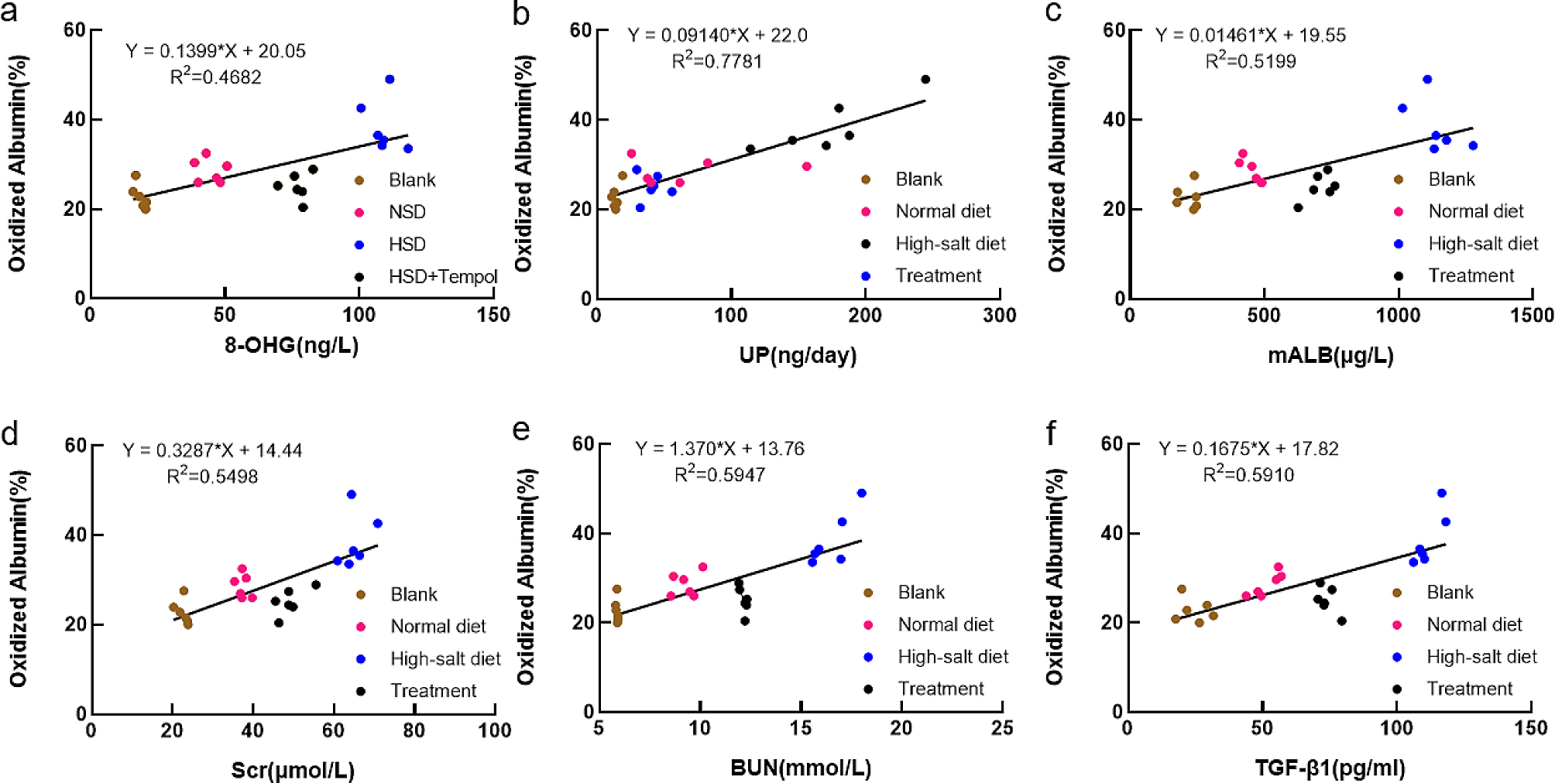
Positive relationship between oxidized albumin and kidney function markers. Correlation between oxidized albumin% and 8-OHG(**a**), urine protein(**b**), mALB(**c**), Scr(**d**), BUN(**e**), serum TGF-β1(**f**). The blank group (*n* = 6), normal diet group (*n* = 6), high-salt group (*n* = 6), and treatment group (*n* = 6) were evaluated

A positive correlation exists between oxidized albumin% and 8-OHG, urinary protein, mALB, Scr, BUN, and serum TGF-β1. (Fig. 7) These results indicate that oxidized albumin% can serve as a novel biomarker with greater sensitivity for assessing oxidative stress compared to the traditional markers in our rat model.

## Discussion

Chronic kidney disease (CKD) stands as a significant global health concern, impacting millions of individuals worldwide [2, 25]. It is a multifaceted and diverse disorder characterized by the gradual decline in renal function over time [26]. Oxidative stress, arising from an imbalance between reactive oxygen species (ROS) production and the body’s antioxidant defense system, has been implicated in the development of different renal diseases, including CKD [27, 28].

Concerning biomarkers for oxidative stress, the escalation in oxidative damage can be discerned through the identification of stable oxidation end products formed via diverse reaction pathways. This challenge arises because detecting circulating reactive oxygen species (ROS) in vivo proves exceptionally difficult due to their heightened biological reactivity and brief half-life [29]. The biomarkers linked to oxidative stress play a pivotal role in assessing the physiological impact of oxidative stress within biological systems. These indicators provide valuable insights into the extent of oxidative damage and the overall redox balance in cells and tissues. Commonly explored biomarkers encompass levels of reactive oxygen species (ROS), concentrations of malondialdehyde (MDA), and the activities of antioxidant enzymes like superoxide dismutase (SOD) and catalase. Monitoring these biomarkers not only advances our comprehension of the oxidative stress status but also holds significance in unveiling potential associations with various health conditions.

Albumin, the main protein in human plasma, is crucial for maintaining colloid osmotic pressure and transporting molecules in the body [30, 31]. However, it is susceptible to post-translational modifications, such as oxidation, which can impair its biological function [32–34]. Oxidized albumin has been recognized as a potential biomarker and contributor to the development of CKD [12, 35, 36]. Recent studies have delved into the correlation between oxidized albumin levels and CKD, yielding promising results. Elevated levels of oxidized albumin have been observed in CKD patients, suggesting its potential involvement in disease progression [36]. Moreover, intervention studies using antioxidants in CKD patients have shown a reduction in oxidized albumin levels. This implies that oxidized albumin could serve as a valuable biomarker and target for therapeutic intervention in CKD patients [12, 13, 37, 38].

In a recent study conducted by Dornas et al., the administration of tempol resulted in a reduction in blood pressure, indicating the involvement of reactive oxygen species (ROS) in the development of elevated blood pressure in 5/6 nephrectomy models [38]. In our study, high-salt diet group exhibited a markable urinary protein elevation compared to normal salt group. In contrast, a decline in urinary protein was observed after tempol administration. Experimental investigations have revealed that Tempol possesses the ability to reduce glomerular barrier permeability through its capacity to eliminate ROS and alleviate oxidative stress, consequently reducing urinary protein leakage [39]. It has also been found that Tempol can regulate the reabsorption and secretion of urinary protein by renal tubular cells, thus affecting urinary protein excretion. In animal models, Tempol administration resulted in a significant decrease in urinary protein levels and a simultaneous improvement in kidney function indicators [17]. Additionally, Tempol also exhibits a regulatory effect on the processes of inflammation and fibrosis, both of which are crucial in urinary protein-related diseases [40]. Tempol inhibits inflammatory cells activation and inflammatory mediators release, thereby reducing inflammation and cellular damage [18, 41]. Moreover, Tempol can also suppress oxidative stress and extracellular matrix proteins synthesis in the fibrotic process, thus reducing renal fibrosis and the progression of urinary protein [42]. Oxidative stress markers, such as oxidized albumin, mALB and 8-OHdG exhibit a similar trend to urinary protein levels. This may be attributed to tempol’s ability to scavenge ROS generated by oxidized albumin, inhibit lipid peroxidation, and prevent the oxidation of albumin molecules [43, 44]. Additionally, Tempol has been shown to inhibit protein carbonyl groups formation, a marker of protein oxidation induced by oxidized albumin [45].

In our experiment, the Treatment group exhibited substantial improvements in final body weight, urine volume, and renal function (serum creatinine, BUN, and proteinuria) in comparison to the High-salt diet group. As expected, the Treatment group exhibited marked glomerulosclerosis and tubulointerstitial damage in the remnant kidney. Importantly, the Treatment group experienced a reduction of more than 50% in both glomerulosclerosis and the extent of tubular injury. The consistent findings observed in rats from the Treatment group are likely attributed to the antioxidant characteristics of the compound under investigation. Notably, the administration of Tempol successfully normalized markers associated with oxidative stress in rats on a High-salt diet. In our study, Tempol administration following a high-salt diet resulted in a significant increase in TGF-β1 levels compared to the group on a normal-salt diet. A previous study has shown that Tempol exerts anti-fibrotic and anti-inflammatory effects by inhibiting the TGF-β1 signaling pathway [46, 47]. Specifically, Tempol can suppress TGF-β1-induced collagen synthesis, matrix metalloproteinase activity, and extracellular matrix remodeling, reducing the expression of extracellular matrix proteins and fibrosis-related genes induced by TGF-β1 [47].

Following high salt loading, there was a significant increase in the secretion of representative pro-inflammatory cytokines TNF-α, IL-6, and MCP-1. Consistent with the cytokine release findings, the mRNA expression levels assessed through RT-qPCR demonstrated the suppressive impact of Tempol in rats from the Treatment group. Immunostaining results indicate the retention of p65 (red fluorescence) in rats subjected to a high-salt diet, while Tempol reverses this effect in the Treatment group. Western blot analysis further demonstrates an elevation of p65 in the High-salt group but a decline in the Treatment group. Corresponding with the immunostaining findings, Tempol administration significantly reduces the translocation of p65 into the nucleus—a critical event in the activation of the NF-κB pathway. Several studies have reported that Tempol can attenuate TNF-α and IL-6-induced inflammation by inhibiting TNF-α and IL-6 production and release in various cell types [48–50]. This effect is achieved through the suppression of NF-κB activation, a major transcription factor involved in TNF-α and IL-6-mediated signaling pathways [51, 52]. Additionally, Tempol has been demonstrated to mitigate TNF-α-induced oxidative stress and alleviate IL-6-induced inflammation and tissue damage in various disease models [53, 54].

Histopathological and immunohistochemical is illustrated in Fig. 3a. Compared to normal diet group rats, glomerular cells were significantly elevated in High-salt diet group rats. Tempol treatment significantly reduced the effect. Renal TGF-β1 and Smad3 mRNA levels were markedly increased in the high-salt diet group relative to the normal diet group rats. However, in the treatment group, renal TGF-β1 and Smad3 mRNA levels were notably decreased compared to the high-salt diet group rats, consistent with the Western blot results. Previous studies have elucidated that Tempol modulates Smad3 signaling, a key mediator of the TGF-β1 pathway [55–57]. Tempol attenuates Smad3 phosphorylation and nuclear translocation, leading to decreased transcriptional activity of profibrotic genes [58, 59]. Furthermore, Tempol impedes the binding of Smad3 to target gene promoters, thereby reducing the synthesis of Collagen I and other extracellular matrix components [60, 61]. Collectively, these findings suggest that Tempol exerts an inhibitory effect on Smad3-mediated fibrosis.

Our investigation systematically explores the potential effects of tempol on oxidative stress biomarkers, covering a range of indicators. This includes assessments of physiological parameters, biochemical testing parameters, morphological and immunostaining analyses, protein expression levels, and the secretion of inflammatory cytokines. The study aims to reveal new insights into the impact of Tempol on specific, both known and unknown, signaling pathways in chronic kidney disease (CKD), with a focus on the regulation of NF-κB and TGF-β1/Smad3 pathways. Additionally, we introduced a pivotal oxidative stress indicator—oxidized albumin—as a potential biomarker, a facet that has been relatively underexplored in CKD-related research. The detection of oxidized albumin provides further insights into the oxidative stress status in CKD, enhancing our understanding of the pathological mechanisms underlying the disease, particularly the interplay between oxidative stress and kidney damage. The observed positive correlation between the percentage of oxidized albumin and a range of biomarkers, including 8-hydroxyguanosine (8-OHG), urinary protein, microalbumin (mALB), serum creatinine (Scr), blood urea nitrogen (BUN), and TGF-β1 in a rat model, indicates that oxidized albumin may serve as a sensitive indicator for oxidative stress. This sensitivity positions it as a potentially invaluable tool for evaluating oxidative stress in analogous experimental contexts.

Our findings demonstrate that Tempol significantly influences the levels of oxidized albumin. This implies its potential in modulating CKD-related oxidative stress, thereby reinforcing support for its clinical application. Should oxidized albumin indeed play a substantial role in CKD, it could serve as a valuable biomarker for disease diagnosis, monitoring treatment effectiveness, or predicting prognosis in the future.

## Conclusion

Our study unveils the promising therapeutic potential of Tempol in preventing and managing kidney disease in the 5/6 nephrectomy rat model. Tempol treatment effectively reduces renal fibrosis, oxidative stress, and inflammation levels. These renoprotective effects suggest a new approach for developing interventions against kidney disease. Moreover, the observed positive correlation between oxidized albumin and other conventional biomarkers indicates the potential utility of oxidized albumin as a metric for assessing oxidative stress in chronic kidney disease.

### Electronic supplementary material

Below is the link to the electronic supplementary material.

Supplementary Material 1

## Data Availability

The datasets utilized and/or analyzed in the present study can be obtained from the corresponding author upon reasonable request.
