# Supplementary material for: Assessing the effects of tempol on renal fibrosis, inflammation, and oxidative stress in a high-salt diet combined with 5/6 nephrectomy rat model: utilizing oxidized albumin as a biomarker
Source: BMC Nephrol. 2024 Feb 23;25:64. doi: 10.1186/s12882-024-03495-0 (PMC10893674; doi:10.1186/s12882-024-03495-0)

Supplementary Material

Assessing the Effects of Tempol on Renal Fibrosis, Inflammation, and Oxidative Stress in a High-Salt Diet combined with 5/6 Nephrectomy Rat Model: Utilizing Oxidized Albumin as a Biomarker

Beibei Liu^1^, Yanling Hu^1^, Bing-Feng Li^1,^ *

1 College of Life and Health, Nanjing Polytechnic Institute, Nanjing, China.

***Correspondence:** Dr. Bing-Feng Li

College of Life and Health, Nanjing Polytechnic Institute, Nanjing, China.

No.188 Xinle Road, Luhe District, Nanjing, Jiangsu, 210048, China

Tel: +86 13814021850

Email: 13814021850@139.com

## Supplementary Figures

Supplemental Figure 1. Original western blots picture of Smad3/GAPDH, TGF-β1/GAPDH, Collagen I / GAPDH.


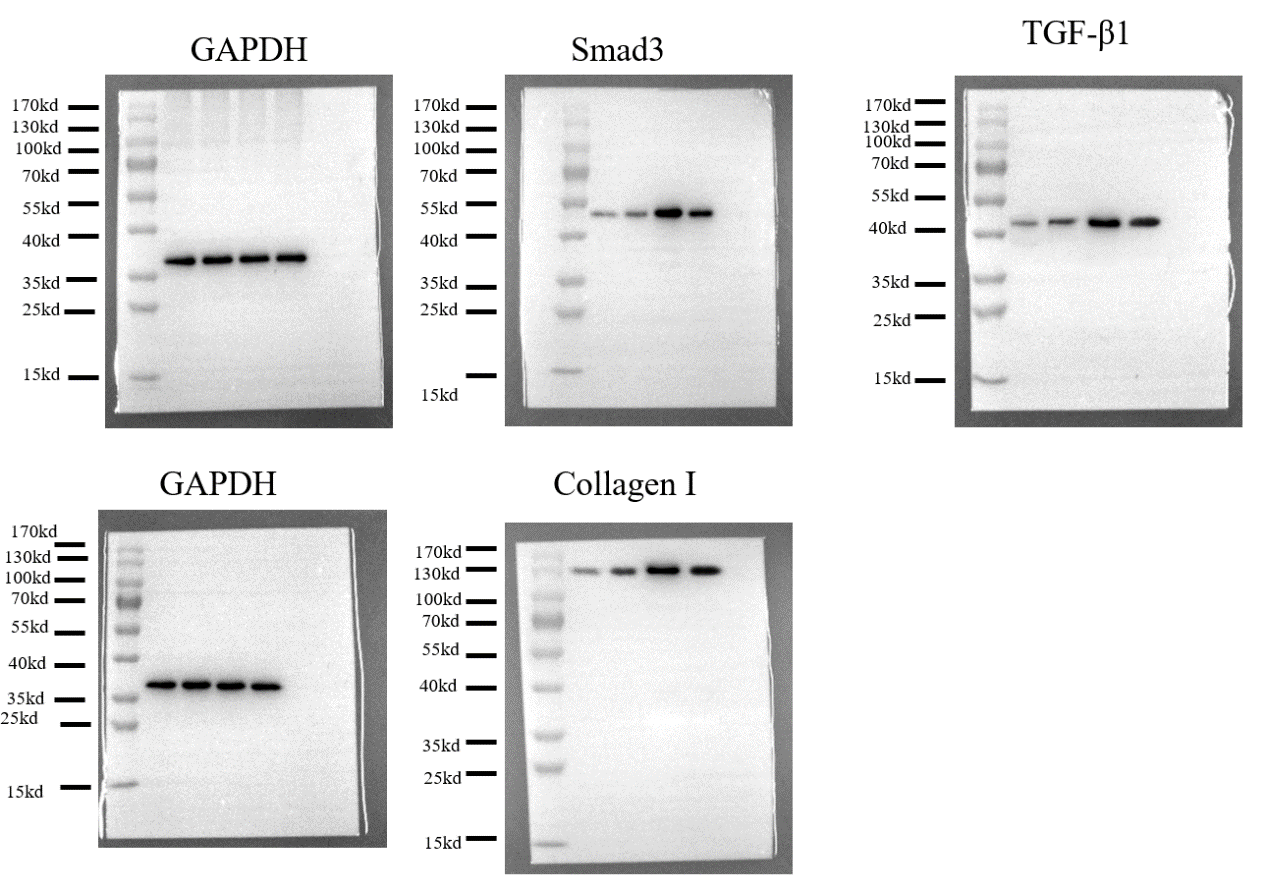


Supplemental Figure 2. Original western blots picture of P65-NF-κB / GAPDH.


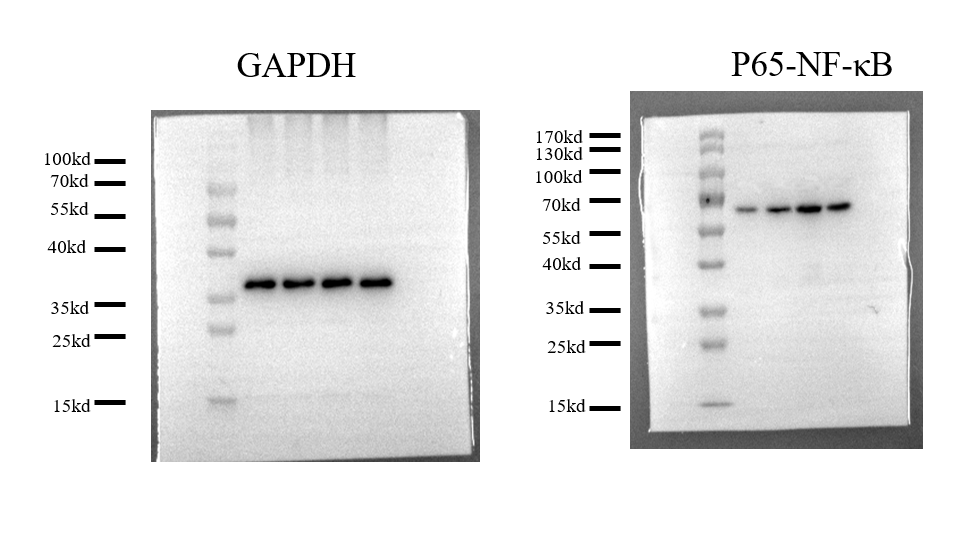

Supplement: Supplementary file 1 — Supplementary Material 1 [file 12882_2024_3495_MOESM1_ESM.docx]
